# Impact of adverse drug reactions on the outcomes of tuberculosis treatment

**DOI:** 10.1371/journal.pone.0269765

**Published:** 2023-02-07

**Authors:** Flávia M. Sant´Anna, Mariana Araújo-Pereira, Carolina A. S. Schmaltz, María B. Arriaga, Bruno B. Andrade, Valeria C. Rolla

**Affiliations:** 1 Postgraduate Program Clinical Research in Infectious Diseases, National Institute of Infectious Diseases Evandro Chagas, Oswaldo Cruz Foundation, Rio de Janeiro, Brazil; 2 Clinical Research Laboratory on Mycobacteria, (LAPCLIN-TB), National Institute of Infectious Diseases Evandro Chagas, Oswaldo Cruz Foundation, Rio de Janeiro, Brazil; 3 School of Medicine, Federal University of Bahia, Salvador, Brazil; 4 Multinational Organization Network Sponsoring Translational and Epidemiological Research (MONSTER) Initiative, Salvador, Brazil; 5 Laboratory of Inflammation and Biomarkers, Gonçalo Moniz Institute, Oswaldo Cruz Foundation, Salvador, Brazil; 6 Curso de Medicina, Escola Bahiana de Medicina e Saúde Pública (EBMSP), Salvador, Brazil; 7 Curso de Medicina, Universidade Salvador (UNIFACS), Laureate Universities, Salvador, Brazil; The Armed Forces Goyang Hospital, REPUBLIC OF KOREA

## Abstract

**Background:**

Adverse drug reactions (ADR) challenge successful anti-tuberculosis treatment (ATT). The aim of this study was to evaluate the impact of ATT-associated ADR and related factors on ATT outcomes.

**Methods:**

A prospective cohort study of persons with tuberculosis (TB) at a referral center in Rio de Janeiro, Brazil, from 2010 to 2016. Baseline information: race, sex, schooling, economic status, tobacco, drugs and alcohol abuse, HIV-infection status and comorbidities were captured during TB screening and diagnosis. Laboratory exams were performed to confirm TB diagnosis and monitor ADRs, favorable (cure and treatment completion) and unfavorable (death, loss to follow up and failure) outcomes were prospectively captured. The Kaplan-Meier curve was used to estimate the probability of ADR-free time. A logistic regression analysis (backward elimination) was performed to identify independent associations with unfavorable outcomes.

**Results:**

550 patients were enrolled, 35.1% were people living with HIV (PLHIV) and ADR occurred in 78.6% of all participants. Smoking (OR: 2.32; 95% CI:1.34–3.99) and illicit-drug use (OR:2.02; 95% CI:1.15–3.55) were independent risk factors for unfavorable outcomes. In PLHIV, alcohol abuse and previous ART use were associated with unfavorable outcomes. In contrast, ADR increased the odds of favorable outcomes in the overall population. PLHIV more frequently experienced grade 3/4-ADR (18.36%), especially “liver and biliary system disorders”. Lower CD4 counts (<100 cells/uL) were associated with hepatotoxicity (p = 0.03). ART-naïve participants presented a higher incidence of ADR in comparison with ART-experienced patients.

**Conclusion:**

Substance use was associated with unfavorable outcomes, highlighting the need for better strategies to reduce this habit. In contrast, ADRs were associated with favorable outcomes. Attention to the occurrence of ADR in PLHIV is essential, especially regarding hepatotoxicity in those with high immunosuppression.

## Introduction

Anti-tuberculosis treatment (ATT) has been available worldwide and can reach more than 90% of effectiveness [1]. However, the challenges in achieving this goal persist as ATT can cause adverse drug reactions (ADR) that lead to increased morbidity and compromise adherence eventually contributing to treatment failure, relapse, or emergence of resistant strains [2, 3].

The ATT recommended by World Health Organization (WHO) and implemented since 2009 in the Brazilian guidelines is a fixed-dose combination (FDC), single-tablet combination of four drugs (rifampicin, isoniazid, pyrazinamide, and ethambutol) daily, for 2 months (intensive phase), followed by daily rifampicin and isoniazid for 4 months (maintenance phase) [4].

Retrospective studies conducted in Brazil found an incidence of ADR between 23% to 83% [5–7] and in a meta-analysis including other non-Brazilian studies, the incidence varied from 8.4% to 83.5% [8]. ADRs are multifactorial [9, 10] and the major determinants are unadjusted prescribed doses of medications, patient’s age, nutritional status, alcohol consumption, altered liver and kidney function and HIV-infection [11, 12].

In people living with HIV (PLHIV), co-administration of antiretroviral therapy (ART) and ATT increases the risk of drug interactions, immunopathological responses, and ADRs [13]. When an ADR occurs, ATT may have to be discontinued for a time, which might ultimately interfere with the outcomes, particularly in those patients with advanced immunodeficiency [3]. In a prospective study conducted in Rwanda, the incidence of serious ADR during ATT was 35%, of which 13% was liver toxicity. In this study, ADRs have been associated with an almost two-fold increased risk of unsuccessful treatment outcomes [3]. In contrast, a Sweden study showed that serious ADRs (23%) increased in PLHIV who initiated ART during ATT but were associated with favorable outcomes [14].

According to data from the Brazilian Ministry of Health, in the year of 2020, 8.5% of new diagnoses of tuberculosis (TB) were in PLHIV [15]. ART during ATT is challenging because of drug-to-drug interactions and increased risk of ADR. In Brazil there are few studies on ADR during ATT [5–7] correlating with outcomes. To the best of our knowledge, there are no studies to date including ADR during ATT in PLHIV. In this regard, the present study aims to evaluate the impact of ATT on ADR and identify factors associated with treatment outcomes in a cohort of patients diagnosed and treated for TB in our center, including PLHIV.

## Methods

### Ethics statement

The Institutional Review Board of the National Institute of Infectious Diseases Evandro Chagas (INI) approved the study (CAAE: 86215118.5.0000.5262). Written informed consent was obtained from all participants involved in the study and all clinical investigations were conducted according to the principles expressed in the Declaration of Helsinki.

### Study design

We conducted a prospective cohort study with TB patients treated and followed up at the Clinical Research Laboratory on Mycobacteria (LAPCLIN-TB) of the National Institute of Infectious diseases Evandro Chagas (INI), Oswaldo Cruz Foundation (FIOCRUZ), Rio de Janeiro, Brazil, from January 2010 to December 2016. INI is a tertiary reference hospital for infectious diseases inside the campus of FIOCRUZ and cares for TB and PLHIV (among other infectious diseases) in two integrated programs. Patients are referred to LAPCLIN-TB by other health units (as well as internal reference) but also come directly to our hospital if they suspect of having TB or other infectious diseases. In LAPCLIN-TB, a cohort study of TB cases has been active since 2000, with data registered at a standardized visit template used to document all visits in an electronic system (CECLIN). The information is captured at each patient’s visit including ADR occurrence and outcomes of TB treatment. In our study, we initially describe the characteristics of patients included and secondly the characteristics of ADR presented by them.

### Inclusion and exclusion criteria

The inclusion criteria were 18 years old or more and pulmonary, extrapulmonary or disseminated TB. The exclusion criteria were death or loss to follow up (LTFU) within the first 15 days of ATT (early death or early LTFU avoiding data capture), rifampicin mono resistance or rifampicin and isoniazid resistance (MDR) and lack of information on outcomes. Patients initially diagnosed with TB who start ATT and were later diagnosed with another disease rather than TB were also excluded.

### TB diagnosis and follow up visits

All patients in LAPCLIN-TB are screened for TB using acid fast smears, Xpert MTB-RIF™ and culture (LJ or MGIT). For positive cultures a Drug Susceptibility Test (DST) is performed for first line TB drugs. Histopathology is also made for tissue biopsy. TB diagnosis could be clinical-radiological (based on symptoms and signs and chest X-ray findings without bacteriological confirmation) or confirmed by laboratory tests (acid fast smears and/or culture and/or histopathological findings and/or Xpert-MTB/RIF™). In cases without laboratory confirmation, a positive therapeutic response to ATT had to occur to consider TB as the proper diagnosis. Clinical-radiological improvement with ATT was considered when TB signs and symptoms (like cough, dyspnea, fever, weight loss, and anorexia) improved with ATT and the radiological images also improved throughout the follow-up. Visits were scheduled at baseline (TB diagnosis and ATT initiation), 15, 30, 60, 90, 120, 150 and 180 days after ATT initiation. Sometimes treatment was prolonged, especially for disseminated or extrapulmonary TB cases due to a guideline recommendation or physician decision. In these cases, there were monthly visits scheduled after the 180 days of ATT. Data were entered into an electronic medical record (CECLIN) and standardized information was collected using a predefined template in all visits.

During the baseline visit, information collected included demographic variables such as age, sex, race (self-reported), marital status, schooling (illiterate, elementary school up to 12 years, and high schooling above 12 years), social behavior (alcohol abuse, tobacco, illicit drugs use), clinical information such as clinical forms of TB (pulmonary, extrapulmonary or disseminated—2 or more non-contiguous sites), presence of comorbidities (self-reported for diabetes, Chronic obstructive pulmonary disease (COPD), hypertension, among others), and tested for HIV, hepatitis B and C. Concomitant medications use was also recorded. At baseline visits, laboratory tests were collected: hemogram, urea, creatinine, uric acid, alanine (ALT) and aspartate (AST) transferases, albumin, alkaline phosphatase, gamma glutamyl transferase (GGT), hepatitis B, C and HIV serology. X-rays were done for diagnosis and at follow up visits. At subsequent visits, ADR were monitored with clinical information and laboratory evaluation (hemogram and biochemistry including liver enzymes) in all visits. For known PLHIV, CD4 count, and HIV viral load (VL) were done at baseline visits and after ART introduction and at the end of ATT (6 months).

### Anti-TB treatment (ATT)

Since 2009, Brazilian guidelines incorporated WHO-recommendation of rifampicin 600 mg, isoniazid 300 mg, pyrazinamide 1600 mg and ethambutol 1100 mg in Fixed Dose Combination (FDC) for two months, followed by four months of rifampicin 600 mg and isoniazid 300 mg in FDC for both new and retreatment TB cases until drug-susceptibility tests were available [4]. For patients weighing between 36 to 50 kg the daily dose was rifampicin 450 mg, isoniazid 225 mg, pyrazinamide 1200 mg and ethambutol 825 mg in the first 2 months followed by rifampicin 450 mg and isoniazid 225 mg until the end of treatment. In some Brazilian basic health unit’s TB treatment is directly observed, but not in our tertiary center [4]. A daily dose of pyridoxine (40–100 mg) was also prescribed to prevent neurotoxicity associated with isoniazid.

### HIV treatment

Each assistant physician, according to Brazilian guidelines for Sexual Transmitted Diseases and AIDS [16], prescribed ART for PLHIV. The recommended ATT for PLHIV included a rifamycin (rifampicin or rifabutin), which was chosen according to the ART regimen appropriate for each specific patient. Patients who used rifampicin were treated with Efavirenz 600 mg/day or Raltegravir 800 mg/day or Dolutegravir 50 mg twice a day. Rifabutin 150 mg/day was chosen when Protease Inhibitors (PI) were prescribed as part of ART [11]. ART was introduced as soon as possible, which happened around the first month of ATT for naïve patients in most cases.

### Smoke habit

A current smoker was defined as someone who was smoking at the time of TB diagnosis and/or at the beginning of ATT or who stopped smoking due to TB symptoms but went back to smoke during ATT.

### Illicit drug users

All patients who reported to be currently using drugs were considered illicit drug users.

### Alcohol abusers

All patients answered the CAGE questionnaire [17] and those who answered yes to 2 or more questions were considered as having significant alcohol addiction.

### Adverse Drug Reaction (ADR)

ADR were defined according to signs and symptoms (clinical), laboratory tests abnormalities (laboratory) or both (clinical and laboratory). ADRs were categorized according to WHO classification for adverse reactions [18]. The scale of intensity of ADR was based on the Division of AIDS table for grading the severity of adult and pediatric ADR [19]: Grade 1: mild event, Grade 2: moderate event, Grade 3: severe event, Grade 4: potentially life-threatening event, Grade 5: death. The detailed description is on the **S1 Table**. The causality of ADRs were assessed using the Naranjo Scale, as improbable, possible, probable, and definitively related to ATT [20]. Only probable, possible, and definitively related ADR were considered for the analysis. According to the time of onset we classified ADR as early, if detected in the first two months of anti-TB treatment (intensive phase) and late if it had started after two months of TB treatment initiation (maintenance phase).

### Outcomes

Outcomes were based on WHO classification [21]. The success of treatment consisted of cure and treatment conclusion which were considered favorable outcomes. Unfavorable outcomes were grouped (composite outcome) and included treatment failure (remaining smear-positive after the 4th month of ATT), transfer to different treatment facilities (patients who were transferred out to other health unit), LTFU (interruption of treatment for two or more consecutive months) and all death associated with TB were revised and the causality accessed. Transfer to a different treatment facility was not an outcome considered in our analysis because no cases occurred in our study.

### Data analyses

Descriptive statistics was performed using the median values with interquartile ranges (IQR) as measures of central tendency and dispersion for continuous variables. Categorical variables were described using frequency (no.) and proportions (%). The Mann–Whitney U test (for two unmatched groups) was used to compare continuous variables and the Pearson chi-square test was used to compare categorical variables between study groups. The Kaplan-Meier curve was used to estimate ADR-free probabilities. Raw data is depicted in S1 File. A logistic binary regression was performed with backward stepwise elimination method. In the first model, we included all the collected variables (age, sex, race, marital status, schooling, social behavior, clinical form of TB), presence of comorbidities, HIV status, hepatitis B and C status, hemogram, urea, creatinine, uric acid, ALT, AST, albumin, alkaline phosphatase, GGT) was performed to identify independent associations between characteristics of TB patients with the composite unfavorable treatment outcome. Only variables that remained in the last model were plotted. Results from regression were presented in terms of point estimates and 95% confidence intervals (CI). All analyses were pre-specified. Differences with p-values below 0.05 after adjustment for multiple comparisons (Holm-Bonferroni) were considered statistically significant.

The statistical analyses and data visualization were performed using rstatix (version 0.4.0), stats (version 3.6.2), ggplot2 (version 3.3.2), survivor (version 3.2.7) and survminer (version 0.4.8) R packages.

## Results

**Fig 1:**
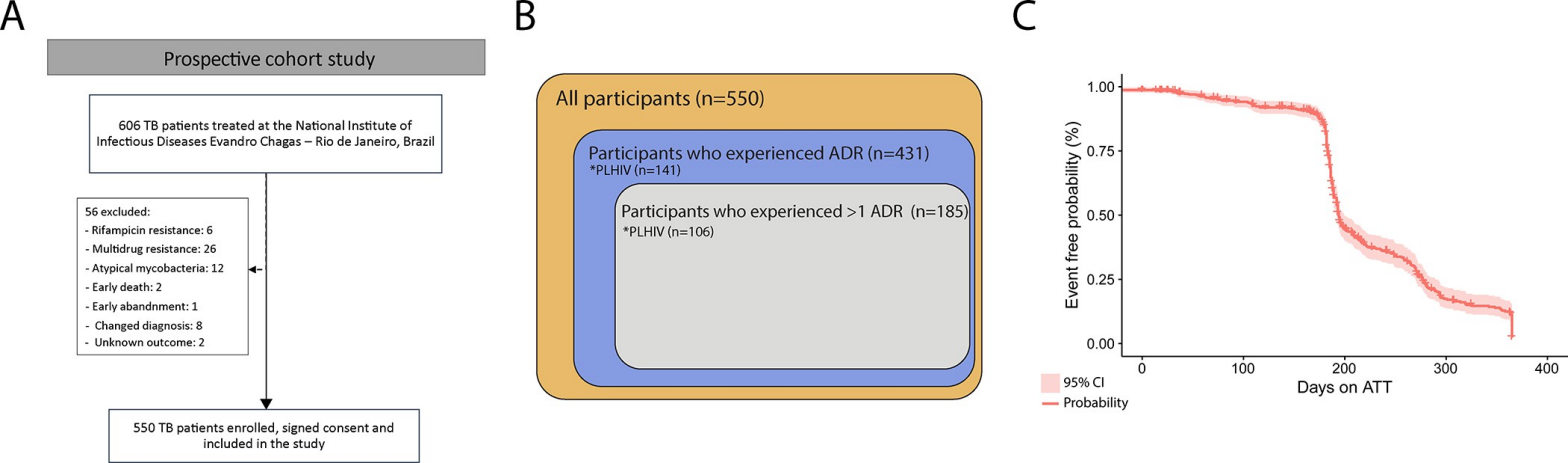
Study design and probability of presenting adverse drug reactions during the antitubercular treatment. (A) Study design. (B) Event free curve (Kaplan-Meier) of tuberculosis patients during antitubercular treatment. Mean time (in state, restricted max time = 365) in days of event occurrence: 140.7. Abbreviation: ADR: adverse drug reaction; ATT: anti-TB treatment; CI: confidence interval; TB: tuberculosis.

From 2010 to 2016, 606 patients started ATT at LAPCLIN-TB Fifty-six patients with exclusion criteria were ruled out: 26 with MDR TB, 6 rifampicin resistance, 20 with other diagnoses during ATT (including non-tuberculous mycobacteria), two due to early death, and one because of early LTFU. Two patients were removed from the dataset due to lack of information on the outcomes. Thus, a total of 550 patients were included in this study **(Fig 1A)**.

**Table 1** shows a comparison of favorable and unfavorable outcomes regarding their sociodemographic and clinical characteristics. The median age was 38 years, 329 (59.8%) were male and 193 (35.1%) were PLHIV. Among patients with unfavorable outcomes: 84 were LTFU, 7 had a treatment failure and 7 died. No cases of transfer were detected in our casuistic.

**Table 1:** Clinical, sociodemographic and comorbidities of tuberculosis patients according to outcomes.

	All patients (n = 550)	Favorable outcomes (n = 452)	Unfavorable outcomes (n = 98)	p value
**Male, n (%):**	329 (59.8)	256 (56.6)	73 (74.5)	**0.002**
**Age, median (IQR):**	38 (29–49)	39 (29–50)	36 (28.2–46.8)	0.138
**Race, n (%):**				**<0.001**
White	281 (51.1)	250 (55.3)	31 (31.6)	
Black/Pardo	269 (48.9)	202 (44.7)	67 (68.4)	
**Schooling, n (%):**				**<0.001**
Illiterate	21 (3.82)	16 (3.54)	5 (5.10)	
Elementary School	449 (81.6)	356 (78.8)	93 (94.9)	
High School	80 (14.5)	80 (17.7)	0 (0.00)	
**Married, n (%):**	279 (50.7)	239 (52.9)	40 (40.8)	**0.040**
**Tobacco use, n (%):**	191 (34.7)	134 (29.6)	57 (58.2)	**<0.001**
**Alcohol abuse, n (%):**	212 (38.5)	156 (34.5)	56 (57.1)	**<0.001**
**Illicit drug use, n (%):**	121 (22.0)	78 (17.3)	43 (43.9)	**<0.001**
**Initial Weight, median (IQR):**	58 (50.6–67.5)	59.7 (50.7–9.6)	56.3 (50.1–64)	**0.023**
**ADR occurrence, n (%):**	431 (78.6)	372 (82.5)	59 (60.8)	**<0.001**
**Type of tuberculosis, n (%):**				**0.023**
PTB	290 (52.7)	238 (52.7)	52 (53.1)	1
EPTB	170 (30.9)	148 (32.7)	22 (22.4)	0.060
Disseminated	90 (16.4)	66 (14.6)	24 (24.5)	**0.025**
**Changed treatment, n (%):**	64 (11.6)	49 (10.8)	15 (15.3)	0.282
**Hepatitis B or C, n (%):**	28 (5.09)	23 (5.09)	5 (5.10)	1
**Diabetes mellitus, n (%):**	50 (9.09)	46 (10.2)	4 (4.08)	0.087
**Hypertension, n (%):**	78 (14.2)	72 (15.9)	6 (6.12)	**0.018**
**COPD, n (%):**	10 (1.82)	9 (1.99)	1 (1.02)	1
**HIV infection, n (%):**	193 (35.1)	141 (31.2)	52 (53.1)	**<0.001**
ART naive, n (%)	74 (38.7)	62 (44.3)	12 (23.5)	**0.015**

Data are shown as median and interquartile (IQR) range or number and frequency (percentage). Data were compared between the clinical groups using the Mann–Whitney U test (continuous variables) or the Pearson’s χ 2 tests (for data on frequency). Bold in p-value indicates p < 0.05.

Weight was measured in kilograms. Abbreviations: ADR: adverse drug reaction; HIV: Human immunodeficiency virus; IQR: Interquartile Range; TB: tuberculosis.

All participants who died (N = 7) were PLHIV with clinical and/or laboratorial signs of advanced immunodeficiency. No deaths were related to ADR. We observed significant differences in race, schooling, smoking habits, and alcohol abuse among cases with favorable and unfavorable outcomes. ADR occurred in 78.6% of all participants, 35.1% of ADR were in PLHIV. Despite the high incidence, occurrence of ADR was higher in the group of participants who experienced favorable ATT outcomes.

As described above, of the 550 patients included in the study only 431/550 (78.4%) patients had at least one ADR. A total of 695 ADR was documented. Our analysis of the ADR severity has shown that 264/695 (38%) ADR were grade 1, 352/695 (50.6%) were grade 2, 67/695 (9.64%) were grade 3 and 12/695 (1.73%) were grade 4. According to the onset of ADR, 592/695 (85.2%) have occurred in the intensive phase of treatment and 103/695 (14.8%) have occurred in the maintenance phase. Of all ADR occurrences, 14/695 (2.1%) were related, 171/695 (24.6%) were probable, and 510/695 (73.4%) possibly related to ATT.

**Fig 2:**
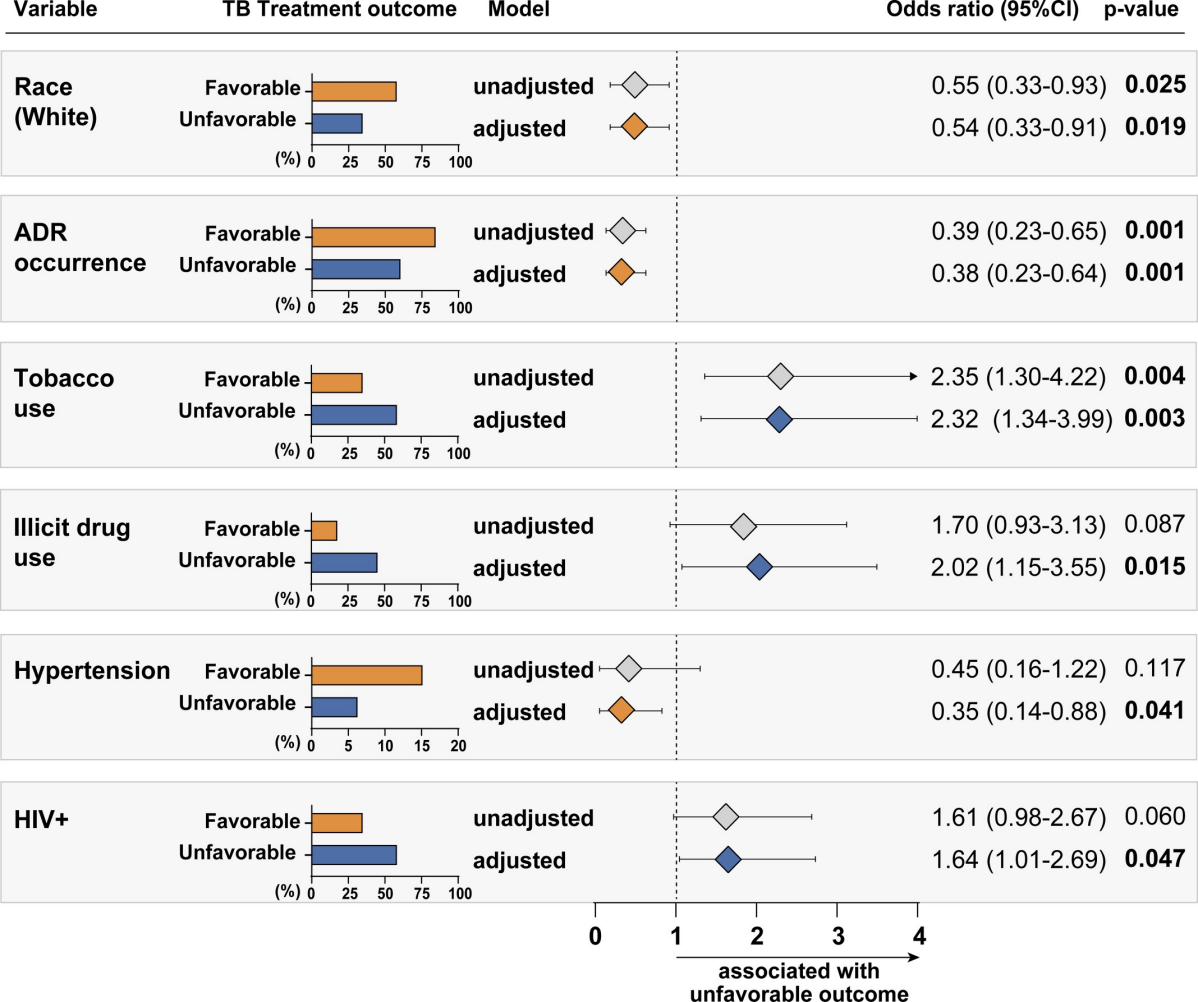
Association between clinical characteristics and tuberculosis treatment outcomes among tuberculosis patients. The logistic binary regression model (backward stepwise regression) was performed to evaluate the independent associations between clinical characteristics, including adverse drug reaction occurrence, of tuberculosis patients and variables showed in the univariate analyses (Table 1) and unfavorable treatment outcome. Only variables remaining in the final model are shown. Abbreviation: ADR: adverse drug reaction; CI: confidence interval; TB: tuberculosis.

Our analysis of the type, severity, date of onset, relationship with the ATT and WHO-ART classification according to outcomes are presented in **Table 2**. According to WHO classification, gastro-intestinal system disorders (23.9%) followed by “metabolic and nutritional disorders” (22.4%), were the most prevalent ADR related to unfavorable outcomes. The probability of ADR was higher until 140 days of ATT **(Fig 1B).** In the logistic regression model, smoking habit, illicit drugs use, and HIV-infection were associated with unfavorable outcomes **(Fig 2).**

**Table 2:** ADR characteristics according to ATT outcome.

	All (n = 695)	Favorable (n = 628)	Unfavorable (n = 67)	p value
**Type of event, n (%):**				**0.95**
**Clinical + Laboratory**	**87 (12.5)**	**78 (12.4)**	**9 (13.4)**	
**Laboratory**	**228 (32.8)**	**207 (33.0)**	**21 (31.3)**	
**Clinical**	**380 (54.7)**	**343 (54.6)**	**37 (55.2)**	
**Adverse reactions duration (days), median (IQR):**	**77.0 (39.0–142)**	**81.5 (43.0–147)**	**44.0 (25.0–85.0)**	**<0.001**
**Severity of ADR, n (%):**				**0.128**
**Grade 1**	**264 (38.0)**	**246 (39.2)**	**18 (26.9)**	
**Grade 2**	**352 (50.6)**	**314 (50.0)**	**38 (56.7)**	
**Grade 3**	**67 (9.64)**	**57 (9.08)**	**10 (14.9)**	
**Grade 4**	**12 (1.73)**	**11 (1.75)**	**1 (1.49)**	
**Onset of ADR, n (%):**				**0.38**
**before 2nd month (early)**	**592 (85.2)**	**532 (84.7)**	**60 (89.6)**	
**after month 2 (late)**	**103 (14.8)**	**96 (15.3)**	**7 (10.4)**	
**Relation of ADR with ATT, n (%):**				**0.601**
**Related**	**14 (2.01)**	**14 (2.23)**	**0 (0.00)**	
**Probable**	**171 (24.6)**	**153 (24.4)**	**18 (26.9)**	
**Possible**	**510 (73.4)**	**461 (73.4)**	**49 (73.1)**	
**WHO ART classification, n (%):**				**0.207**
**Central and peripheral nervous system disorders**	**45 (6.47)**	**43 (6.85)**	**2 (2.99)**	
**Gastro-intestinal system disorder**	**151 (21.7)**	**135 (21.5)**	**16 (23.9)**	
**Liver and biliary system disorders**	**54 (7.77)**	**44 (7.01)**	**10 (14.9)**	
**Metabolic and nutritional disorders**	**171 (24.6)**	**156 (24.8)**	**15 (22.4)**	
**Musculo-skeletal disorders**	**63 (9.06)**	**56 (8.92)**	**7 (10.4)**	
**Other**	**46 (6.62)**	**45 (7.17)**	**1 (1.49)**	
**Skin and appendages disorders**	**125 (18.0)**	**113 (18.0)**	**12 (17.9)**	

Data are shown as median and interquartile (IQR) range or number and frequency (percentage). Data were compared between the clinical groups using the Mann–Whitney U test (continuous variables) or the Pearson’s χ 2 tests (for data on frequency). Bold in p-value indicates p < 0.05.

We next compared the clinical characteristics and ADR presentation of patients according to HIV status. Of note, six patients who have presented ADR were removed due to lack of HIV status information, remaining 425/431 patients (141 PLHIV and 284 non-HIV). Comparing these groups, we have noted that there were significant differences between the groups **(Table 3).** Self-reported race, marital status, smoking habits, alcohol abusers, illicit drug use in the PLHIV group were associated with unfavorable outcomes. ADR occurred in 78.6% of total participants and in 33,1% of PLHIV. Although viral hepatitis (B and C N = 21) serology was included in our model, it was not associated with unfavorable outcomes, but the number of positive cases was small, and we did not test hepatitis VL to confirm active hepatitis. The main finding of this investigation was that PLHIV experienced more severe forms of ADR than non-HIV participants, especially liver and biliary system disorders.

**Table 3:** Sociodemographic and clinical characteristics of patients who presented ADR according to HIV status.

	All patients with ADR (n = 425)	PLHIV (n = 141)	non-HIV (n = 284)	p value
Male, n (%):	258 (60.6)	97 (68.8)	161 (56.5)	**0.019**
Age, median (IQR):	39.0 (29.0–50.0)	37.0 (31.0–47.0)	40.0 (28.0–53.0)	0.244
White (race), n (%):	230 (54.0)	52 (36.9)	178 (62.5)	**<0.001**
Married, n (%):	229 (53.8)	57 (40.4)	172 (60.4)	**<0.001**
Tobacco use, n (%):	144 (33.8)	64 (45.4)	80 (28.1)	**0.001**
Alcohol abuse, n (%):	160 (37.6)	70 (49.6)	90 (31.6)	**<0.001**
Illicit drug use, n (%):	88 (20.7)	44 (31.2)	44 (15.4)	**<0.001**
Type of TB, n (%):				**<0.001**
PTB	222 (52.1)	65 (46.1)	157 (55.1)	
EPTB	135 (31.7)	19 (13.5)	116 (40.7)	
Disseminated	69 (16.2)	57 (40.4)	12 (4.21)	
Initial Weight, median (IQR):	59.0 (51.0–68.0)	59.6 (50.7–68.0)	57.1 (53.8–64.0)	0.836
Hepatitis B or C, n (%):	21 (4.93)	11 (7.80)	10 (3.51)	0.091
Diabetes mellitus, n (%):	41 (9.62)	11 (7.80)	30 (10.5)	0.47
Hypertension, n (%):	70 (16.4)	12 (8.51)	58 (20.4)	**0.003**
COPD, n (%):	8 (1.88)	1 (0.71)	7 (2.46)	0.28

Data are shown as median and interquartile (IQR) range or number and frequency (percentage). Data were compared between the clinical groups using the Mann–Whitney *U* test (continuous variables) or the Pearson’s χ 2 tests (for data on frequency). Bold in p-value indicates p < 0.05.

Weight was measured in kilograms. Abbreviations: COPD: Chronic obstructive pulmonary disease; ART: Antiretroviral Therapy; HIV: Human immunodeficiency virus; IQR: Interquartile Range; TB: tuberculosis.

Although IRIS is not an ADR in this study, among PLHIV, 9 cases of IRIS were diagnosed. IRIS is not frequent and was not diagnosed in association with ADR in our study.

According to clinical data, it was observed that among PLHIV, median CD4 count was 166 cells/μl and those with ADR grade 3 and 4 had lower CD4 count compared to those with ADR grade 1 and 2 and most of those with ADR grade 3/4 displayed CD4 count below 100 cells/uL **(Table 3)**. The characteristics of ADR differed between cases of grade 1/2 compared to those graded 3/4 in terms of type, time of onset, relationship with treatment, and affected system. The most affected system in patients with grade 3/4 ADR was the liver and biliary system **(Table 4)**. Considering all PLHIV, 53.1% experienced unfavorable outcomes (p<0.001). Among them, 183 (33.3%) were ART-experienced while 38.7% ART naïve. In the group of ART experienced patients, 48 (49%) presented unfavorable outcomes (p<0.001).

**Table 4:** Clinical and laboratory characteristics according to intensity of ADR in PLHIV.

	All ADR in PLHIV (n = 140)	Intensity of ADR1/2 (n = 114)	Intensity of ADR 3/4 (n = 26)	p value
Type of TB, n (%):				**<0.001**
PTB	65 (46.1)	55 (48.2)	10 (38.5)	
EPTB	19 (13.5)	16 (14.0)	3 (11.5)	
Disseminated	57 (40.4)	43 (37.7)	13 (50.0)	
HIV Viral Load, median (IQR):	4.53 (3.26–5.33)	4.44 (3.21–5.40)	4.86 (3.55–5.21)	0.871
CD4 (cells/uL), median (IQR):	166 (43.0–353)	176 (52.8–360)	110 (26.8–180)	0.065
CD4 counts, n (%):				**0.033**
<100	56 (40.0)	43 (37.8)	13 (50.0)	
100–200	30 (21.4)	21 (18.4)	9 (34.6)	
>200	54 (38.6)	50 (43.9)	4 (15.4)	
Initial Weight, median (IQR):	59.0 (51.0–68.0)	59.6 (50.7–68.0)	57.1 (53.8–64.0)	0.836
Hepatitis B or C, n (%):	11 (7.86)	11 (9.65)	0 (0.0)	0.091
Diabetes mellitus, n (%):	10 (7.14)	7 (6.1)	3 (11.5)	0.47
Hypertension, n (%):	10 (7.14)	8 (7.01)	2 (7.7)	1.000
COPD, n (%):	1 (0.71)	1 (0.88)	0 (0.0)	1.000

Data are shown as median and interquartile (IQR) range or number and frequency (percentage). Data were compared between the clinical groups using the Mann–Whitney U test (continuous variables) or the Pearson’s χ 2 tests (for data on frequency). Bold in p-value indicates p < 0.05.

Weight was measured in kilograms. Abbreviations: COPD: Chronic obstructive pulmonary disease; ART: Antiretroviral Therapy; HIV: Human immunodeficiency virus; IQR: Interquartile Range; TB: tuberculosis.

## Discussion

The impact of unsuccessful ATT can directly impair the strategies for TB control [22, 23]. Although many patients were lost to follow-up in our study, an important finding was that those who had ADR were less likely to have unfavorable outcomes, including being lost to follow-up. This is not what was expected and suggests that close monitoring of ADR influences the outcomes of patients on TB treatment. PLHIV were expected to have more ADR than HIV-unexposed participants, however this was not observed in our study, although they have had higher ADR severity when compared to HIV-unexposed participants. Serious events were mainly liver and biliary system disorders, especially in those patients with lower CD4 counts. This finding is important to emphasize that blood tests should be performed in PLHIV, especially in the first month of TB treatment, to detect potential ADRs and prevent their progression and the need for hospitalization. Physicians should be aware of the possibility of severe ADR in these patients.

In our study, patients were attended by different physicians who in general conduct the whole treatment and have laboratory exams available to help ADR detection and management. Also, medical care is available at our hospital every day, 24 hours a day, which helps ADR management any time they occur.

As expected, most patients who were LTFU had alcohol abuse or illicit drug use/addiction. These findings are of great importance for planning strategies to decrease harm and increase early ART initiation, even with the risk of drug-to-drug interaction. As there was a considerable LTFU (15,2%), representing the predominant unfavorable outcome, it is urgent to create policies to increase treatment adherence.

In our data, male sex was found to be significantly associated with unfavorable outcomes. A possible explanation is that men were less likely to regularly follow treatment when compared with women in our setting [15]. Being highly exposed to tobacco, alcohol abuse, illicit drugs, higher HIV prevalence might contribute to unfavorable outcomes in male sex. In our study, low schooling, smoke habit and illicit drug use were associated with unfavorable outcomes. However, among PLHIV, alcohol abuse was the only independent factor associated with unfavorable outcomes. Other studies in the literature have shown the same correlation between alcohol abuse, [2, 24, 25] and smoke habit associated with unfavorable outcomes, as well as in our study [26].

ADRs were not associated with unfavorable outcomes, contrary to our primary expectations. Probably, patients who experienced ADR in our setting were better managed and/or more closely monitored, which contributed to feeling well cared for and having a good adherence to therapy. In contrast, a previous prospective study in Rwanda with TB patients, with or without HIV-infection, has shown that ADRs were associated with an almost two-fold increased risk of LTFU [2].

Another correlation observed in our study was that high schooling participants did not present any unfavorable outcome, which was not a surprise since they probably have a better understanding about the importance of treatment adherence for TB cure. Definitive treatment interruption before cure can lead to recurrence, development of drug resistance, increased costs, and a worse epidemiologic situation [27].

We also explored the liver and biliary system disorders in PLHIV with advanced immunosuppression and a high incidence of hepatotoxicity was associated with low CD4 count, mainly in those with less than 100 cells/uL. This important finding should help the management of TB in PLHIV, since more attention will be directed to this type of ADR in this population. Most ART naïve patients are diagnosed with HIV due to TB, as HIV serology is done in all patients with TB diagnosis who accepted to be tested [11]. These are, in general, the most severe cases and hospitalization is sometimes necessary not only for TB diagnosis but also to manage severe and serious ADR after ATT introduction. The early ART onset in these cases is of special interest due to the added risk of hepatotoxicity. Sometimes, when ADR occurs, it is necessary to stop all drugs and reintroduce one by one to identify the causality. Many studies have found that PLHIV using concomitant ART and ATT had a higher frequency of ADR and low adherence to treatment [27–29]. In our study we found 26% of PLHIV developing unfavorable outcomes. ART-naïve patients presented a higher incidence of ADR in comparison with ART-experienced PLHIV. Most of these patients were already cared for at the study hospital or came from other health clinics with a history of low adherence to ART and non-attendance to medical appointments. These patients are different from ART-naïve patients that had a recent TB diagnosis and were tested for HIV for the first time.

In our routine care, ART commencement occurs as soon as possible in the first month of TB treatment for those with low CD4 counts, as well as cotrimoxazole prophylaxis, since this approach has already been proved to reduce mortality [30]. Although all those challenges reported here, our TB lethality was relatively low in PLHIV which shows that the strategy of early ART start works, and ADR can be successfully managed.

Paradoxical immune reconstitution inflammatory syndrome (IRIS) was not included in our analysis because IRIS is not an ADR, it is an adverse event and adverse events were not the objective of this paper. IRIS is not very frequent in Brazil like in other regions such as Africa and India [31]. Our group published other papers showing the low prevalence of IRIS in Rio de Janeiro [32–34].

Our study had limitations: directly observed treatment (DOT) was not performed in our site due to our characteristics of being a tertiary hospital and far from most patients’ houses. Viral hepatitis (B and C) was screened with serology and no VL was performed; the low proportion of viral hepatitis in our study limited the analyses in this group. Concerning PLHIV, the proportion of these participants in our casuistic did not allow us to explore ADR associated with each of the antiretroviral regimens. The highest frequency of LTFU also was a limitation, bearing in mind that this did not allow us to assess the impact of ADR on biological ATT outcomes. Finally, our data cannot be extrapolated to other TB sites due to our characteristics of being a tertiary reference hospital (not a clinic), a research institution, with a better structure than many other hospitals in Rio de Janeiro.

The identification of factors associated with unfavorable outcomes could allow health care to appropriately risk-stratify patients for closer management and improve outcomes. In addition, investments in programs destined to reduce tobacco, alcohol abuse and drug addiction are also necessary to improve TB treatment adherence and decrease LTFU. On the other hand, an investment in education is necessary by the government to make patients better understand the disease and the importance of treatment completion.

In conclusion male sex, low schooling, smoke, and illicit drug use were all independently associated with unfavorable outcomes during ATT for all patients with TB. In PLHIV, alcohol abuse and previous ART use were factors associated with unfavorable outcomes. A high incidence of hepatotoxicity in PLHIV was associated with low CD4 count, mainly in those with less than 100 cells//uL. In addition, 26% of PLHIV developed unfavorable outcomes and ART-naïve presented a higher incidence of ADR in comparison with ART-experienced patients, ADR was more frequent in the intensive phase of TB treatment and was not associated with unfavorable outcomes.

## Supporting information

S1 Table Estimating severity grade for adverse drug reactions according to Division of AIDS (DAIDS), 2017. (DOCX)

S1 File Raw data used to generate the results of the study. (CSV)
