# Supplementary material for: Impact of adverse drug reactions on the outcomes of tuberculosis treatment
Source: PLoS One. 2023 Feb 7;18(2):e0269765. doi: 10.1371/journal.pone.0269765 (PMC9904486; doi:10.1371/journal.pone.0269765)
Supplement: S1 Table — (DOCX) [file pone.0269765.s001.docx]

**S1 Table.** Estimating severity grade for adverse drug reactions according to Division of AIDS (DAIDS), 2017.

| **PARAMETER** | **GRADE 1 (MILD)** | **GRADE 2**  **(MODERATE)** | **GRADE 3 (SEVERE)** | **GRADE 4 (POTENTIAL LIFE-THREATENING)** |
| --- | --- | --- | --- | --- |
| Clinical adverse event NOT identified elsewhere in the grading table | Mild symptoms causing no or minimal interference with usual social & functional activities with intervention not indicated | Moderate symptoms causing greater than minimal interference with usual social and functional activities with intervention indicated | Severe symptoms causing inability to perform usual social and functional activities with intervention or hospitalization indicated | Potentially life-threatening symptoms causing an inability to perform basic self-care functions with intervention indicated to prevent permanent impairment persistent disability, or death |

Source: DAIDS (2017).

**Reference:**

Division of AIDS (DAIDS). Table for Grading the Severity of Adult and Pediatric Adverse Events. U.S. Department of Health and Human Services, National Institutes of Health, National Institute of Allergy and Infectious Diseases, Division of AIDS. Version 2.1. (2017).
